# Alterations in Functional Connectivity Measured by Functional Magnetic Resonance Imaging and the Relationship With Heart Rate Variability in Subjects After Performing Orgasmic Meditation: An Exploratory Study

**DOI:** 10.3389/fpsyg.2021.708973

**Published:** 2021-11-11

**Authors:** Andrew B. Newberg, Nancy A. Wintering, Chloe Hriso, Faezeh Vedaei, Marie Stoner, Reneita Ross

**Affiliations:** ^1^Department of Integrative Medicine and Nutritional Sciences, Thomas Jefferson University, Philadelphia, PA, United States; ^2^Department of Radiology, Thomas Jefferson University, Philadelphia, PA, United States; ^3^Department of Obstetrics and Gynecology, Thomas Jefferson University, Philadelphia, PA, United States

**Keywords:** blood oxygen level dependent (BOLD), functional connectivity, fMRI, meditation, sexuality, brain imaging

## Abstract

**Background:** We measured changes in resting brain functional connectivity, with blood oxygen level dependent (BOLD) functional magnetic resonance imaging (fMRI), associated with a creative meditation practice that is augmented by clitoral stimulation and is designed to not only achieve a spiritual experience but to help individuals manage their most intimate personal relationships. Briefly, the meditative state is attained by both the male and female participants while the male stimulates the woman’s clitoris. The goal of this practice, called orgasmic meditation (OM), according to the practitioners is not sexual, but to use the focus on clitoral stimulation to facilitate a meditative state of connectedness and calm alertness between the two participants.

**Methods:** fMRI was acquired on 20 pairs of subjects shortly following one of two states that were randomized in their order – during the OM practice or during a neutral condition. The practice is performed while the female is lying down on pillows with the clitoris exposed. During the practice, the male performs digital stimulation of the clitoris for 15 min. Resting BOLD image acquisition was performed at completion of the practice to assess changes in functional connectivity associated with the performance of the practice.

**Results:** The results demonstrated significant changes (*p* < 0.05) in functional connectivity associated with the OM compared to the neutral condition. For the entire group there was altered connectivity following the OM practice involving the left superior temporal lobe, the frontal lobe, anterior cingulate, and insula. In female subjects, there was altered connectivity involving the cerebellum, thalamus, inferior frontal lobe posterior parietal lobe, angular gyrus, amygdala and middle temporal gyrus, and prefrontal cortex. In males, functional connectivity changes involved the supramarginal gyrus, cerebellum, and orbitofrontal gyrus, cerebellum, parahippocampus, inferior temporal gyrus, and anterior cingulate.

**Conclusion:** Overall, these findings suggest a complex pattern of functional connectivity changes occurring in both members of the couple pair that result from this unique meditation practice. The changes represent a hybrid of functional connectivity findings with some similarities to meditation based practices and some with sexual stimulation and orgasm. This study has broader implications for understanding the dynamic relationship between sexuality and spirituality.

## Introduction

Sexuality has long been associated with creativity and spirituality. Some traditions find ways of incorporating sexuality or sexual stimulation into an overall spiritual approach such as the Taoist tradition that describes sexual practices designed to bring the Yin and the Yang (the feminine and the masculine) into harmony. In the Tantric tradition that is part of Hinduism, sexual energy and the excitement generated in the sacral chakra in the pelvic region, is used to raise “spiritual energy.” The practices of yoga meditation, and some type of Buddhism, also integrate aspects of sensuality and meditation. A number of prior studies have examined the effects of acute meditation practices on brain physiology and functional connectivity (Fox et al., 2016; Hernández et al., 2018; Devaney et al., 2021). Some of these have been activation studies evaluating brain function during the meditation practice or a task affected by the practice. Others have explored changes in resting brain function as the result of the immediate or long term effects of a given practice.

With this background in mind, it seems appropriate within this special topic issue to consider a more scientific analysis of the relationship between sexual stimulation and spiritual experience. We had the opportunity to explore the neurophysiological effects of a creative meditation method that involves female clitoral stimulation with a partner (for this study a separate male partner was chosen by each female participant). It is important to note that according to the practitioners, both the male and female participants are engaged in the meditation practice and both the female as well as the male have intense meditative or even spiritual-like experiences such as the feeling of oneness or connectedness, and feelings of intense awareness. Emotional reactions are also reported and can include a profound sense of relaxation, energy, or joy.

It is a well-defined practice, lasting a specified 15 min, with a few minutes of preparation before the practice and a brief concluding part after the practice (see below for additional details). This practice, called orgasmic meditation (OM) is thought to produce intense meditative states including a sense of connectedness, energy, and positive emotions. Here, we studied the effects of OM on resting state functional connectivity measured within approximately 1 hour of the practice. Although this practice is called, OM, by the practitioners, the goal is not specifically to achieve sexual orgasm or climax. Importantly, the practice is not performed for sexual gratification but rather specifically for meditative purposes using clitoral stimulation to facilitate a meditative experience. This is confirmed by the subjective descriptions of the experience by the practitioners which do not use words related to sexuality but rather words such as energy, unity, or awareness.

This point about the meditative aspect of the practice is an important one, particularly as it relates to defining a “meditation practice.” There are many ways of defining and characterizing what a meditation practice actually is and how it is structured and performed (Nash et al., 2013). An important part of many types of meditation practices is focusing on specific physiological processes or activities such as breathing or walking. Thus, applying the principle of attentiveness or mindfulness to any physical activity arguably can become a form of meditation. If one accepts that these physical activities could be incorporated into forms of meditation, it seems reasonable to potentially include one that involves sexual arousal. In OM, practitioners describe a number of feelings that are common to other forms of meditation. Thus, an important question is whether this particular practice is neurophysiological similar or different compared to other types of practices.

Over the past 20 years, a number of meditative and spiritual practices have been studied using functional magnetic resonance imaging (fMRI) by our group and others (Newberg et al., 2001, 2010a; Wang et al., 2011). This research has helped to better understand which brain structures and functional networks are involved in these practices. Structures that have been associated with meditative practices include the frontal lobes, parietal lobes, and limbic structures. More recent studies have also determined that larger networks such as the salience network and default mode network (DMN) are involved in meditative practices. A number of other studies have demonstrated how different meditation programs affect emotion, cognition, and other brain processes (Afonso et al., 2020). However, more research is needed in order to assess the many different types of meditative and spiritual practices, and their effects on the brain.

In order to observe neurophysiological changes in both the female and male participants, we decided to use fMRI with resting blood oxygen level dependent (BOLD) to measure resting functional connectivity. These measures were performed shortly following the practice since the practice itself cannot be performed in the scanner environment due to the required positions and movements of the participants. However, it is hoped the results reflect brain changes associated with the impact of the recent performance of the practice on the resting state of the brain (Miall and Robertson, 2006). While this is not ideal for studying changes specifically occurring during the practice, the findings should shed some light on how these practices affect the brain. Further, there are a number of other studies that have explored the effects of meditation practices and spiritual experiences indirectly either due to similar challenges scanning the brain during the actual practices or to assess longer term effects that result from performing practices over many years (Kemmer et al., 2015; Eyre et al., 2016; Smith et al., 2020). In addition, if the OM practice were to result in functional connectivity changes after the practice, the findings would have implications for potential future therapeutic applications of this technique that could change brain regions over longer periods of time (Tambini and Davachi, 2013).

We compared the functional connectivity shortly after both the OM state and a “neutral” state in which the participants were in the same room, positioned the same way, and even performing a sensory stimulation of the leg rather than the clitoris. Thus, all aspects of the practice in the neutral condition were matched with the exception of the clitoral stimulation and the meditation element. We also randomized the order of these two conditions which were done on two separate days.

Based on the current literature, and the study design, we tested the following questions and hypotheses: (1) What brain regions are significantly affected by the OM practice that are related to other meditation practices? We hypothesized a number of brain regions would have altered functional connectivity including the limbic areas associated with emotional processing, the basal ganglia associated with the reward network, frontal regions involved with attention, and posterior regions involved with the DMN (see also below). Each of these areas have been implicated in other neuroimaging studies of meditation techniques. For example, several of our studies, in addition to others, have found altered frontal lobe function during concentrative meditation techniques (Herzog et al., 1990–1991; Newberg et al., 2001; Newberg et al., 2010a). Frontal lobe function appears to increase during practices that involve attentional focus and decrease during practices involving a sense of surrender or sense of flow (Newberg et al., 2001, 2006; Ulrich et al., 2016). Since the male subjects do report subjectively a feeling of flow, “getting lost,” or “absorbed” in the practice, we might expect altered functional connectivity associated with the frontal lobe. Regarding the females, we might observe similarly fewer changes in functional connectivity of the frontal lobes since they are recipients of the stimulation, and thus, are not performing an attentional task. Another general area we have observed to be affected during intense meditation practices is the parietal lobe (Newberg et al., 2001; Khalsa et al., 2009). Decreased activity in the parietal lobe has been associated with a loss of the sense of self and feelings of self-transcendence (Newberg et al., 2001; Newberg and Iversen, 2003; Urgesi et al., 2010). We predicted that there would be altered functional connectivity with the parietal lobes during the OM practice since they describe similar experiences.

Although the OM practice is considered a meditation by the practitioners, it does involve stimulation of the clitoris, and hence, the second question we investigated was: (2) What brain regions are significantly affected by the OM practice that are related to sexual stimulation? We hypothesized that there would be the brain changes associated with OM that might correspond to those more specifically reported with clitoral stimulation. There are a number of fMRI and PET scan studies that have explored manual clitoral stimulation, particularly during orgasm. The literature on clitoral and/or sexual stimulation has shown a mixture of findings based on the specific results of the stimulation (e.g., for sensory reception, pleasure, and orgasm). For example, significant increases in the sensory cortex and the inferior parietal lobe were found when clitoral stimulation was compared to rest (Georgiadis et al., 2006). However, when females progress to orgasm there tend to be significant decreases in cerebral blood flow (CBF) in the left lateral orbitofrontal cortex, inferior temporal gyrus and anterior temporal pole. During orgasm in both men and women, studies have found activation in the cerebellum, anterior cingulate gyrus, and dopaminergic pathways (Komisaruk et al., 2004).

Overall, in terms of brain processes themselves, we expected that the OM practice would resemble meditation based practices more so than practices that only involve sexual arousal or orgasm. Since clitoral stimulation is used to augment the meditative state, we hypothesized that the pattern of brain activity would be unique with elements distinguishing it from both sexual experience as well as meditative practices. Given the manner in which the practice is performed, with one person giving the stimulation (the male) and one person receiving the stimulation (the female), there would be unique differences between the males and females related to the performance of the practice and the specific aspects related to sexual stimulation.

Another aspect of meditation practices, as well as sexual stimulation, are effects on the autonomic nervous system. We have previously developed a model of meditation practices that include a complex interaction between sympathetic and parasympathetic activity (Newberg and Iversen, 2003). Recent studies of meditation practices have shown marked changes in heart rate and heart rate variability. Specifically, there seems to be increased heart rate variability associated with different meditation practices. In a similar manner, sexual stimulation is associated first with a sense of arousal and increased sympathetic activity followed eventually by parasympathetic activity during orgasm itself (Calabrò et al., 2019). Given these effects in both meditative practices and sexual stimulation, we asked: (3) Will the OM practice be associated with significant changes in heart rate variability measures? We hypothesized that there would be specific autonomic changes as measured by heart rate variability associated with the OM practice. Thus, we measured several heart rate parameters before, during, and after the OM practice and the neutral practice.

In addition, we asked: (4) Will changes in functional connectivity between certain brain areas correlate with changes in autonomic function? We hypothesized that if the OM practice was more similar to sexual stimulation, brain areas more specific to sexual stimulation and arousal such as the hypothalamus, limbic system, basal ganglia, and cerebellum, might be particularly involved. And if the OM practice more specifically represents a meditation practice, then there would be correlations between higher cortical areas and autonomic activity.

Finally, we asked: (5) Will there be changes unique to the males and females separately and how will such changes relate to those of the group evaluated in its entirety? We hypothesized that there should also be a different set of unique changes when evaluating the entire group as a single cohort (i.e., evaluating the males and females together) that would be more consistent with various types of meditative experiences including feelings of oneness, connectedness, or awareness. Thus, we hypothesized that for the women, there would be specific alterations in functional connectivity between the parietal and frontal lobe regions associated with the sensory clitoral stimulation. Further, we hypothesized that there would be changes in functional connectivity associated with the limbic areas, dopamine areas, and the thalamus. Since there is no sexual stimulation at all in males, we hypothesized that they would have altered connectivity associated with the frontal regions consistent with their self-described feelings of “flow.” We anticipated that there would be alterations in the functional connectivity of the DMN including the parietal regions and posterior cingulate cortex. Finally, for all subjects, we expected potential changes in functional connectivity in the DMN along with the social areas of the brain including the fusiform gyrus, angular gyrus, and supramarginal gyrus. More recent research has also implicated the cerebellum in a number of cognitive and emotional processes, including meditation practices, and hence we expected functional connectivity between the cerebellum and various cortical and limbic structures. Based on these hypotheses, we focused our analysis on the above described regions with the goal of determining the pattern of functional connectivity associated with this enhanced form of meditation.

## Materials and Methods

### Subjects

Study subjects, both male and female were enrolled through the Marcus Institute of Integrative Health at Thomas Jefferson University Hospital. Forty candidates including 20 females and 20 males (age: 39.0 ± 10.1) and 21 males (40.8 ± 9.7) participated in this study. Subjects were recruited between September 26, 2019 and June 30, 2021. Written informed consent, approved by the Institutional Review Board of Thomas Jefferson University, was obtained from all subjects. Subjects were not compensated for participation in the study but were provided travel expenses. Subjects were included in the study if they were healthy individuals who had been performing the OM practice for at least 1 year on a regular basis (defined as at least 2–3 times per month). Since the OM practice is performed in pairs, female subjects were enrolled first and then they selected their male partner. Their male partner had to be someone with whom they had done the practice before and who subsequently agreed to participate. Subjects who were married were included but only if they performed the practice with their spouse. Subjects were excluded from the study if: (1) they had any physical, neurological, or psychological disorders that might affect cerebral function; (2) they were taking medications that may alter cerebral function; or (3) they were unable to lie still in the scanner. Female subjects of childbearing potential were excluded if they were pregnant or breastfeeding. No subjects dropped out of the study once they were enrolled.

### Orgasmic Meditation Practice

There were two test subjects, a male (giver) and a female (receiver) who performed the practice together in a closed room by themselves in a private area of the imaging facility. The male was clothed the entire time and did not receive any sexual stimulation. The female was clothed except to allow exposure of her genitalia. The room was prepared with a blanket and pillows on the floor according to the standard OM practice methods. The female would then lie down on the pillows with the male who was giving the meditation stimulus seated by the receiver’s right side. The male performed stimulation of the clitoris for 15 min, which is performed while using a sterile glove and lubricant as needed. There was no sexual intercourse or penetration, and hence, no risk of pregnancy or sexually transmitted diseases due to the method of the practice.

Prior to beginning the meditation, the two participants communicate with each other that the practice is about to begin. They then begin and a timer is set for 15 min. The timer was set by the participants as well as the research team. When the practice was completed, the subjects rested for 15 min and then were escorted into the scanner one at a time fully clothed. The female of each pair was scanned first for every scanning session. MRI images were acquired as described below on a 3T Siemens mMR PET–MRI scanner (Siemens Medical Solutions USA, Inc., Malvern, PA, United States) over the course of approximately 30 min. Overall, because of the consistency of the timing of the practice, we were able to maintain similar delays in the scan acquisition timing. Thus, within each of the gender groups the timing on scan acquisition was on average within approximately 15 min. However, the females were scanned on average 31 min after completing either the OM or neutral condition (this delay was planned for the FDG PET scans). The males were then scanned on average 77 min after completing either condition. We will discuss the implications of the different timing delays in section “Discussion.”

A neutral, comparison state was also performed with each of the subjects to account for the specific elements of the practice without the actual meditation or clitoral stimulation component. Thus, subjects entered the meditation room and were positioned in the same manner as during the actual meditation practice. The male was asked to gently stroke the female’s upper leg to account for the motor activity in the male and the sensory response in the female. They performed this neutral condition for the same time period of 15 min. After the 15 min neutral condition, the subjects rested for 15 additional minutes and then were brought into the scanner. Importantly, the order of the meditation and neutral conditions was randomized with half the pairs doing the OM meditation first and half doing the neutral condition first.

### Heart Rate Evaluation

In order to measure heart rate and heart rate variability, we used the HeartMath system (Boulder Creek, CA, United States). This is a commercially available device that uses an earlobe clip monitor to assess heart rate and calculate a variety of heart rate and heart rate variability parameters based on the measures. In order to obtain an adequate signal, we acquired heart rate data over 5 min periods. This was performed at baseline (pre), during the practice time itself, and then immediately following the practice (post). The 5 min of heart rate data obtained during the practice were measured in the final 5 min of the 15 min practice which is the most intense part of the practice.

Once the heart rate data was collected, the heart rate measures were automatically evaluated by the HeartMath software to correct for motion and other artifacts. The data were also viewed visually by the principal investigator to confirm that there were no artifacts that were missed by the automated system. Although we did not specifically track respirations, which are known to potentially affect heart rate variability we did use the following techniques to reduce any influence of respiratory variation – (1) we instructed subjects to sit quietly with eyes open and breathe normally during the 5 min data collection; (2) the HeartMath software automatically de-artifacts for anomalous data points; and (3) we observed for non-normal breaths and removed those timepoints. Once the data was de-artifacted, the software automatically calculated a variety of heart rate variability measures based on a power spectral analysis that provides data on distinct frequency bands (Shaffer and Ginsberg, 2017). Since our goal was to focus on vagal tone and heart rate variability, we primarily looked at the square root of the mean squared difference of successive N–N intervals (RMSDS), as well as the high frequency variability which is specifically associated with vagal tone. These measures were obtained for the pre-, during-, and post-, time points for each subject and for each condition – the OM practice and the neutral practice. These values could then be analyzed as continuous variables for the entire group, as well as for both males and females separately. Analysis of the heart rate measures were tested using a two-way analysis of variance with the group and state as the two factors. Furthermore, these continuous variables could be compared to functional connectivity data obtained after both the OM practice and the neutral practice conditions. Specifically, the change of values for the heart rate measures pre- and post-the neutral and OM conditions were used as additional variables to be analyzed with respect to the change in functional connectivity measures described below.

### MRI Acquisition, Analysis, and Statistics

#### MRI Acquisition

Participants underwent fMRI after the OM session and after the control session in a similar manner using the following sequences. All scans were performed using a head coil with a head holder along with the use of foam and cushions which are designed to limit head motion. A T1 MPRAGE sequence was used to perform a high resolution anatomical image to better examine cortical and subcortical volumes. Next, a resting state BOLD scan was collected using an echo planar imaging (EPI) sequence to examine intrinsic functional connectivity of the brain regions. The following imaging parameters were used: FOV = 23.6 cm, voxel size = 3 mm × 3 mm × 4 mm, TR = 2.0 s, TE = 30 ms, slice thickness = 4 mm, number of slices = 34, number of volumes = 180, and acquisition time = 366 s. During rs-fMRI the subjects were instructed to close their eyes, keep their heads still, and rest quietly without thinking about anything in particular for 5 min.

#### MRI Image Processing

Structural and functional data were processed and analyzed using the MATLAB R2020b based functional connectivity toolbox ‘‘CONN toolbox’’ version 18b along with Statistical Parametric Mapping (SPM 12)^1^ (Whitfield-Gabrieli and Nieto-Castanon, 2012). All scans are visually observed prior to processing in order to assess for significant acquisition or motion artifact. The general preprocessing pipeline included slice timing correction, motion correction, functional realignment and unwarping, structural and functional centering to (0, 0, 0) coordinates, structural segmentation and normalization to Montreal Neurological Institute (MNI) space, functional normalization to EPI template in CONN, co-registration of structural and functional data, and spatial smoothing with a smoothing kernel of 6 mm FWHM. In addition, Artifact Detection Toolbox (ART) was set to the 97th percentile setting with the mean global-signal deviation threshold set at *z* = ± 5 and the subject-motion threshold set at 0.9 mm. The artifact detection implemented in CONN was utilized to detect framewise displacement (FD). The computed motion parameters were then used to exclude the outliers. Any volumes which exceeded a motion threshold of 2 mm (translation) and 1° rotation, or more, were excluded. After preprocessing, by applying linear regression and a band-pass filter of 0.008–0.09 Hz, data were denoised to remove the effects of low and high frequency oscillations such as scanner drift, head motion, heart rate, and respiration rate. Then, the anatomical component-based, noise correction strategy (aCompCor) for spatial and temporal processing was used to remove physiological noise factors from the data. This method extracted principal components from white matter and CSF time series and used them as confounds during the denoising step (Behzadi et al., 2007). The implementation of aComCor along with the quantification of subject motion and the identification of outliers through (ART) allows for enhancement of specificity, sensitivity, and validity of first- and second-level connectivity analysis (Whitfield-Gabrieli and Nieto-Castanon, 2012; Shirer et al., 2015).

#### Functional Connectivity Analysis

After the pre-processing is complete, first-level and second-level region of interest (ROI-to-ROI) analyses were performed to measure functional connectivity maps. ROI-to-ROI analyses are Fisher *z*-transformed bivariate correlations between brain regions’ BOLD time-series that quantify associations in the activation at rest and serve as a proxy for connectivity. The time series are calculated by averaging voxel time series across all voxels within each ROI. ROIs are defined and extracted using combined FSL Harvard–Oxford and AAL atlases. Resting state functional connectivity was calculated using two sided bivariate correlations. Next, significant resting state functional connectivity differences among subjects were evaluated between the acquisitions after the OM and neutral conditions after correction for multiple comparisons using the false discovery rate (FDR) method with a threshold set at *p* < 0.05.

All the ROI regions used in the analysis are in the CONN toolbox and are separated as left and right as specified in section “Results” (Table 2) in which we have identified which laterality has had an effect. In general, the toolbox provides a series of default and pre-defined ROIs that were loaded automatically for brain parcellation for cortical, subcortical, and cerebellar areas from the FSL Harvard–Oxford Atlas.

**TABLE 1:** Demographic information about the participant groups.

Variable	Females (*N* = 20)	Males (*N* = 20)
Mean age (±SD)	39.0 ± 10.1	40.8 ± 9.7
Mean years of experience (±SD)	7.8 ± 3.4	5.5 ± 1.9
Mean intensity of the current experience (±SD)	6.1 ± 1.2	7.1 ± 1.5
Mean heart rate during OM practice	71.0 ± 10.3	76.4 ± 9.4
Mean heart rate during neutral practice	63.5 ± 9.3	68.7 ± 9.4

**TABLE 2:** Comparison between the neutral and OM conditions for all subjects, the males separately, and females separately, showing the significant differences in functional connectivity between selected structures and the uncorrected p value (p-Uncor.) and False Discovery Rate corrected *p*-value (p-FDR).

Analysis units	Statistics	*p*-Uncor.	*p*-FDR
**All subjects meditation vs. neutral**			
Left superior temporal – right inferior frontal cortex	*T* = 4.95	0.0000	0.0021
Left superior temporal – left inferior frontal gyrus	*T* = 3.61	0.0009	0.0238
Left superior temporal – right insula	*T* = 4.38	0.0001	0.0049
Left superior temporal – right supplementary motor cortex	*T* = 3.89	0.0004	0.0158
Left superior temporal – left anterior cingulate cortex	*T* = 3.65	0.0008	0.0238
Right superior temporal – right insula	*T* = 4.16	0.0002	0.0096
Right inferior frontal gyrus – vermis	*T* = 4.09	0.0002	0.0352
Left superior temporal – left middle temporal	*T* = −3.81	0.0005	0.0405
**Female subjects meditation vs. neutral**			
Left cerebellum – right thalamus	*T* = 5.08	0.0001	0.0151
Right inferior frontal – right posterior parietal cortex	*T* = 4.62	0.0002	0.0402
Right inferior frontal – right angular gyrus	*T* = 4.25	0.0005	0.0441
Left temporal fusiform cortex – right supplementary motor cortex	*T* = 4.76	0.0002	0.0295
Right amygdala – right middle temporal gyrus	*T* = 5.41	0.0000	0.0077
Right prefrontal cortex – left prefrontal cortex	*T* = −5.14	0.0001	0.0132
**Male subjects meditation vs. neutral**			
Left supramarginal gyrus – left cerebellum	*T* = 5.47	0.0000	0.0038
Left supramarginal gyrus – right lingual gyrus	*T* = 4.95	0.0001	0.0062
Right orbital frontal cortex – right occipital cortex	*T* = 4.73	0.0001	0.0208
Left parahippocampal gyrus – left cerebellum	*T* = −4.65	0.0002	0.0250
Left inferior temporal gyrus – right anterior cingulate	*T* = −5.40	0.0000	0.0045

We compared functional connectivity between the post OM and neutral state for males alone, females alone, and the entire group together. The reason for these three separate comparisons are based upon our initial hypotheses. Since the OM practice involves the participation of two individuals performing distinct but related components, it is reasonable to consider whether the males and females experience different neurophysiological changes. However, it is also of interest to determine whether there are changes that are shared among the participants. Such changes might reflect common aspects of the shared meditative experience during OM.

#### Clinical Measures

In order to assess the effect of the OM practice in a research setting, we asked subjects to describe how they would characterize the experience of the OM practice during the study in terms of intensity and similarity to their usual practice. Thus, we asked all subjects to rate their qualitative experience by answering the following question:

Scale Q1: “How similar was the process you completed to what you have typically known Orgasmic Meditation to be? (on a scale from 0, not at all, to 10, identical/indistinguishable)”.
Scale Q2: “How intense was the practice you just had? (on a scale from 0, not at all intense, to 10, the most intense OM experience you ever had; 5 would be your average experience)”.

## Results

Table 1 shows the demographic information for the participants including mean age, experience, and the subjective scores that the participants provided regarding the quality of the meditation experience. For the entire group, the OM practice during the study was rated as a mean of 9.7 ± 0.6 (out of 10) in terms of its similarity to their usual OM experience. With regard to the intensity of the experience, it was rated 6.6 ± 1.4 (out of 10) for the entire group. The breakdown by males and females is given below.

**FIGURE 1:**
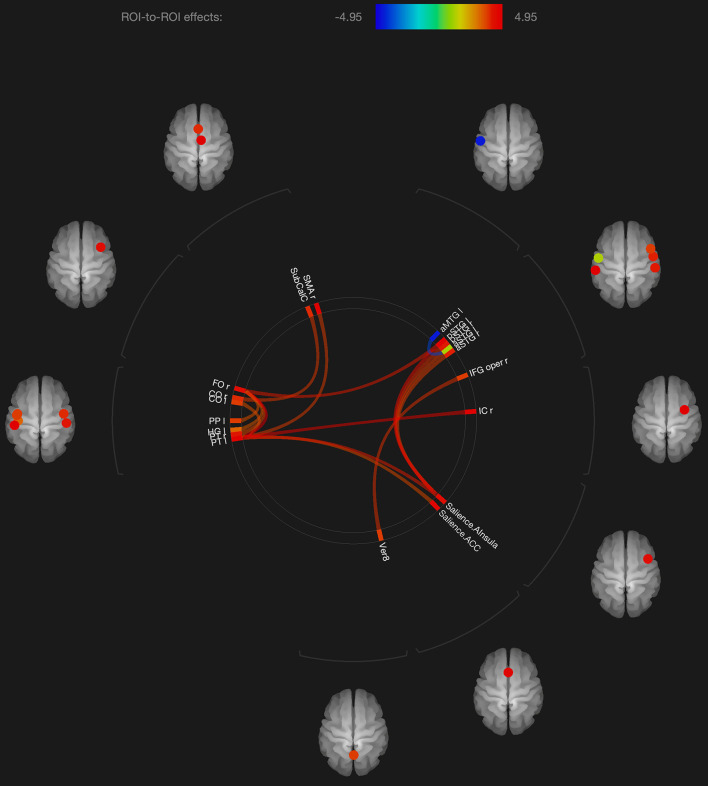
Visualization of ROI-to-ROI connections comparing orgasmic meditation (OM) and neutral groups among all the participants, *p*-FDR corrected <0.05.

**FIGURE 2:**
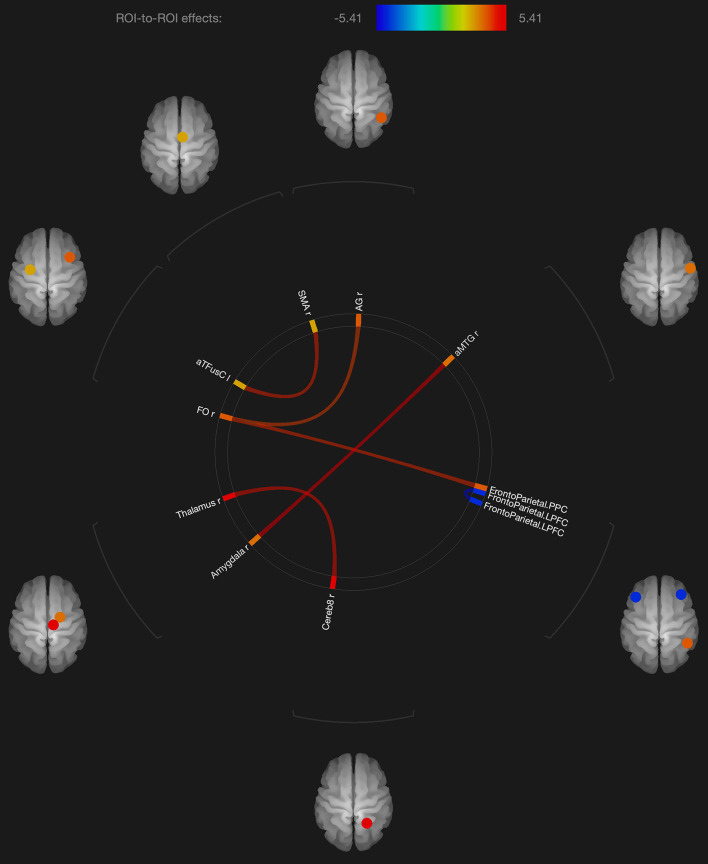
Visualization of ROI-to-ROI connections comparing orgasmic meditation (OM) and neutral groups among females, *p*-FDR corrected <0.05.

**FIGURE 3:**
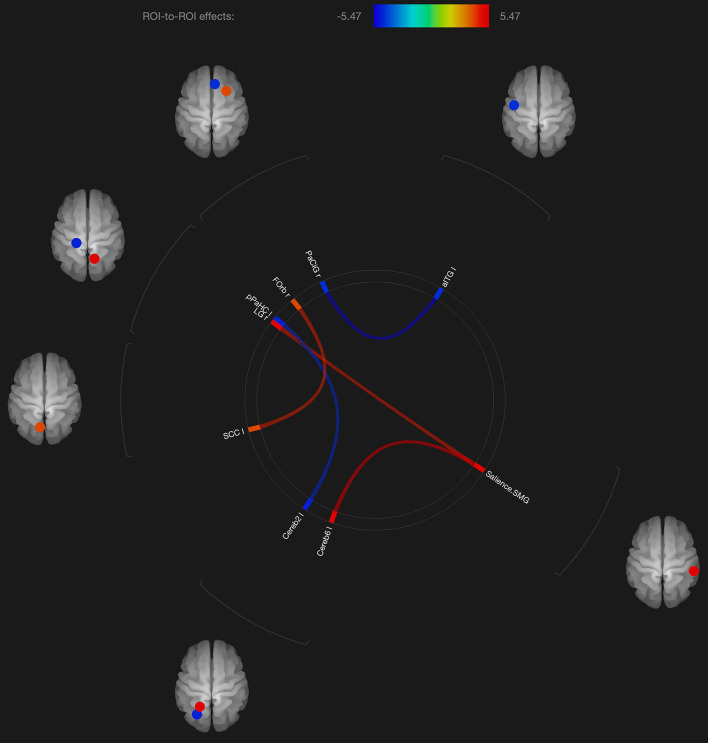
Visualization of ROI-to-ROI connections comparing orgasmic meditation (OM) and neutral groups among males, *p*-FDR corrected) <0.05.

Regarding the fMRI analysis, there were significant differences in functional connectivity observed between the scans obtained shortly after the neutral and OM conditions for the group as a whole, and for the males and females separately. These changes are shown in Table 2 and Figures 1–3. Changes in functional connectivity were also compared to the intensity of the meditative experience for the entire group as well as the males and females separately (see Table 3).

**TABLE 3:** Significant correlations between the change in functional connectivity (OM vs. neutral condition) and the subjective feeling of intensity during the OM practice showing the uncorrected p value (p-Uncor.) and False Discovery Rate corrected *p*-value (p-FDR).

Analysis units	Statistics	*p*-Uncor.	*p*-FDR
**All subjects intensity vs. functional connectivity**			
Right middle frontal gyrus – anterior cingulate/salience	*T* = 4.48	0.0001	0.0112
Right middle frontal gyrus – right Heschl’s gyrus	*T* = 3.79	0.0005	0.0445
Right superior frontal gyrus – right Heschl’s gyrus	*T* = 3.93	0.0004	0.0445
Right cerebellum – left dorsal attention/inferior parietal	*T* = 4.00	0.0003	0.0482
Anterior cerebellum – right nucleus accumbens	*T* = −4.03	0.0003	0.0433
**Female subjects intensity vs. functional connectivity**			
Left cerebellum – anterior cingulate/salience	*T* = 4.68	0.0002	0.0406
**Male subjects intensity vs. functional connectivity**			
Right cerebellum – precuneus	*T* = 4.10	0.0006	0.0419
Right cerebellum – default mode/posterior cingulate	*T* = 4.00	0.0008	0.0419
Left superior temporal gyrus – left default mode/medial prefrontal cortex	*T* = 4.23	0.0005	0.0245
Left temporal pole – left default mode/medial prefrontal cortex	*T* = 4.23	0.0005	0.0371
Left superior temporal gyrus – left supramarginal gyrus	*T* = −4.29	0.0004	0.0245
Left inferior temporal gyrus – right Heschl’s gyrus	*T* = −4.41	0.0003	0.0491
Default mode/medial prefrontal cortex – precuneus	*T* = −3.86	0.0010	0.0427

The results from the heart rate analysis showed that there was an expected significant increase (*p* < 0.001) in heart rate during the OM condition in both males (mean increase +7.7 ± 6.6 bpm or 11.2%) and females (mean increase +7.6 ± 8.6 bpm or 12.0%). This finding supports the general success in performing this stimulatory practice and observing changes in heart rate associated with that practice. Additional results of the analysis of heart rate measure changes demonstrated that, in males, the RMSSD decreased significantly from 47.3 ± 17.7 in the neutral condition to 39.5 ± 21.6 during the OM condition (*p* = 0.026). The RMSSD was also significantly decreased when comparing the value after the neutral condition (48.7 ± 21.6) to the value after the OM condition (42.6 ± 18.2, *p* = 0.021). Interestingly, in the females the RMSSD did not change substantially between the OM and neutral conditions.

In the high frequency power, the males and females had similar results. In males, there was significantly decreased high frequency power during the OM condition (4.34 ± 1.03, *p* = 0.001) compared to the neutral condition (4.88 ± 0.82). However, there was no significant difference between the post-OM or post-neutral condition. In females, there was also significantly decreased high frequency power during the OM condition (4.88 ± 0.98, *p* = 0.03) compared to during the neutral condition (5.21 ± 0.98), and similar to the males, there was no significant difference between the post-OM or post-neutral condition.

With regard to the comparison between functional connectivity and heart rate variability measures, we found a number of significant correlations for the entire group as well as the males and females separately (see Table 4).

**TABLE 4:** Correlation between functional connectivity differences and heart rate measures (RMSSD and high frequency power) showing the uncorrected p value (p-Uncor.) and False Discovery Rate corrected *p*-value (p-FDR).

Analysis units	Statistics	*p*-Uncor.	*p*-FDR
**All subjects change in RMSSD vs. change in functional connectivity**			
Right nucleus accumbens – right caudate	*T* = 4.20	0.0002	0.028
Left parahippocampal gyrus – left precentral gyrus	*T* = −4.20	0.0002	0.028
**Female subjects change in RMSSD vs. change in functional connectivity**			
Left thalamus – right fusiform cortex	*T* = 5.00	0.0001	0.0215
Left default mode – right superior frontal gyrus	*T* = −4.60	0.0003	0.0478
Right dorsal attention – left putamen	*T* = −4.59	0.0003	0.0491
Vermis – left hippocampus	*T* = −5.20	0.0001	0.0143
**Male subjects change in RMSSD vs. change in functional connectivity**			
Right lateral PFC – left central opercular cortex	*T* = −5.32	0.0001	0.0092
Right supplementary motor cortex – right lateral PFC	*T* = −4.56	0.0003	0.0227
Right cerebellum – left planum temporale	*T* = −4.80	0.0002	0.0272
Left precentral gyrus – left parahippocampal gyrus	*T* = −4.52	0.0003	0.0496
**All subjects change in high frequency power vs. change in functional connectivity**			
Left inferior frontal gyrus – left temporal pole	*T* = 4.20	0.0002	0.0283
Left inferior frontal gyrus – right superior temporal gyrus	*T* = 3.76	0.0006	0.0424
Left inferior frontal gyrus – left frontal orbital cortex	*T* = 3.68	0.0008	0.0424
Vermis – right superior temporal gyrus	*T* = −4.45	0.0001	0.0138
Right lateral PFC – right Heschl’s gyrus	*T* = −4.84	0.0000	0.0042
**Female subjects change in high frequency power vs. change in functional connectivity**			
Vermis – left lateral occipital cortex	*T* = 4.60	0.0003	0.0487
Vermis – left superior temporal gyrus	*T* = 4.75	0.0002	0.0356
Left cerebellum – left middle temporal gyrus	*T* = 4.65	0.0003	0.0432
Right cerebellum – left central opercular cortex	*T* = 4.66	0.0003	0.0212
Right lateral PFC – right putamen	*T* = −6.03	0.0000	0.0029
Left amygdala – right angular gyrus	*T* = −6.37	0.0000	0.0015
Right superior temporal gyrus – right hippocampus	*T* = −4.71	0.0002	0.0381
Right superior temporal gyrus – left hippocampus	*T* = −4.30	0.0006	0.0356
Right superior temporal gyrus – left lateral occipital cortex	*T* = −4.22	0.0007	0.0356
Right superior temporal gyrus – left inferior frontal gyrus	*T* = 4.01	0.0010	0.0375
Right lateral occipital cortex – right lateral PFC	*T* = 4.17	0.0007	0.0438
Left angular gyrus – right thalamus	*T* = 4.74	0.0002	0.0364
**Male subjects change in high frequency power vs. change in functional connectivity**			
Left cerebellum – right posterior parietal cortex	*T* = 4.23	0.0006	0.0914
Right cerebellum – right middle temporal gyrus	*T* = −4.18	0.0006	0.0995
Right cerebellum – left superior temporal gyrus	*T* = −3.87	0.0012	0.0995

*Note that a decrease in RMSSD indicates an increase in sympathetic tone so that a positive T score represents increased connectivity associated with decreased sympathetic tone and negative *T* score represents increased connectivity associated with increased sympathetic tone.*

## Discussion

This is the first study that has utilized resting BOLD fMRI to evaluate the functional connectivity changes associated with a unique meditative practice involving clitoral stimulation as a paired practice between two individuals. The goal of the OM practice is to utilize stimulation as the attentional focus of the meditation. The practitioners have described various spiritual-like experiences such as the feeling of oneness or connectedness, as well as a sense of flow and awareness. Emotional reactions including a profound sense of relaxation, energy, or joy are also described. Determining the underlying mechanistic effects of this practice can have important implications for understanding the broader relationship between spirituality and sexuality, as well as creativity.

As described in the introduction, there are several elements of this practice that make it an interesting target for scientific study. It is a well-defined meditation practice that has clearly defined elements and timing. The standardization of the practice makes it easier to develop a consistent research protocol. Other meditation practices can last for hours and can have more open ended approaches such as focusing on a mantra with little other specific guidance as to the approach. The practice is performed in pairs, but the subjects interact in a similar manner each time. The OM practice specifies that both members of the pair are engaged in the meditation practice, even though their role in the practice is different. Since both participants perform different elements of the meditation practice, it is expected that there would be distinctions in their subjective response and their physiology.

Given these aspects of the practice, we attempted to determine if there were specific changes in resting brain functional connectivity that resulted from its performance. We evaluated whether the changes in brain functional connectivity would be comparable to findings in studies of other meditative practices, but could also have some similarities to clitoral stimulation itself (questions #1 and #2). Thus, we hypothesized that brain regions previously found to be associated with meditative practices, through both activation studies and studies of the immediate and long term effects of those practices, would similarly be involved in the OM practice including the frontal lobe, insula temporal lobe, limbic regions, basal ganglia, thalamus, and parietal lobe. We also hypothesized that there would be distinct patterns of functional connectivity changes in both the male and female partners, although we might also expect to see some similarities since they are performing the practice together and both the givers and receivers have reported similar experiences.

The results are consistent with our general hypotheses. Overall, when the entire group was compared between the meditation and neutral condition, there were a number of areas that had significantly different resting functional connectivity. As a whole group, there were significant increases in functional connectivity particularly between the left superior temporal lobe and the left and right inferior frontal, right insula, right supplementary motor cortex and anterior cingulate gyrus, associated with the OM practice. There were also significant increases in the functional connectivity between the right superior temporal lobe and right insula and the right inferior frontal gyrus and the vermis of the cerebellum. These changes ultimately represent the temporal dependency of neuronal activity patterns in anatomically distinct brain regions. Such findings in the period immediately following a practice imply some degree of a lasting effect of the practice on brain function even when the person is no longer performing the practice.

In considering such changes, the superior temporal lobe is part of the DMN’s dorsal medial subsystem which has been shown to be particularly involved in processes like intercessory prayer that is associated with thinking about others. The functional connectivity of this structure that is significantly altered by the OM practice is consistent with the strong social aspect of the paired practice, and also the importance of thinking about, or connecting with, the other individual in the pair. It is interesting that much of the changes in functional connectivity appear to be associated with the left superior temporal gyrus which is more associated with language and memory processes. The left superior temporal gyrus becomes more functionally connected with the inferior frontal cortex which has been involved in several meditation based practices during the actual practice (Davanger et al., 2010).

The increased connectivity between the right superior temporal region and the right insula is consistent with studies evaluating compassion as a long term effect of loving-kindness meditation (Lutz et al., 2008). These practices elicit strong feelings of bonding and connection between people during the practice as, although not in the direct manner as the OM practice. The insula, in conjunction with the superior temporal lobe, appears to be particularly involved in feelings of strong emotional social bonds. The insula also plays a key role in integrating external sensory information, that might be perceived during the OM practice, with emotional awareness. Similarly, the insula has been found to be significantly altered in meditators compared with non-meditators in studies comparing trait characteristics between these groups (Jang et al., 2018).

One fMRI study during yoga meditation revealed that deep stages of meditation are associated with significant activation in the right middle temporal cortex and right inferior frontal cortex that were both observed to be affected by the OM practice (Telles, 2014). Another meta-analysis of 78 meditation studies using functional neuroimaging reported increased activity in the insula, supplementary motor cortices, and anterior cingulate cortex, and prefrontal cortex during meditation practices (Fox et al., 2016), all of which are areas that were found to have their functional connectivity significantly affected by the OM practice.

As expected in our hypothesis to our initial question #5, there was also a distinct pattern in the functional connectivity changes when evaluating the males and females independently. In the females, we observed several regions having significantly different functional connectivity after the OM practice compared to the neutral condition. Specifically, there were significant increases in functional connectivity between the right inferior frontal lobe and the posterior parietal cortex and angular gyrus, the latter two being part of the DMN which has been shown to be altered in other meditation practices (Andrews-Hanna et al., 2014).

More recent meditation studies have focused on the DMN that becomes deactivated during a variety of meditative states. The DMN is characterized by the synchronous activation of several separated regions in the brain, including the medial prefrontal cortex, posterior cingulate cortex, precuneus, inferior parietal lobule, and inferolateral temporal cortex (Raichle et al., 2001). For example, Brewer et al. (2011) investigated the impact of focused attention meditation (i.e., concentration), open monitoring meditation (choiceless awareness), and loving-kindness meditation (a member of the constructive family) on the DMN, showing that, in the three types of meditations, the main nodes of the DMN were deactivated in experienced meditators as a characteristic trait of their brain function. The authors also found a strong coupling between the posterior cingulate, dorsal anterior cingulate, and dorsolateral prefrontal cortices, regions involved in self-monitoring and cognitive control during meditation. While some publications indicate that open monitoring meditation diminishes DMN activity (Garrison et al., 2015), it has also been reported that it results in an increased activation of the precuneus, a DMN hub, in contrast to focused awareness (Manna et al., 2010). In the current study, we did not see widespread changes in the DMN, but a relatively small number of structures that were affected. Such findings might have implications for future studies exploring how the OM practice is associated with various elements of spiritual experiences.

We also observed changes in functional connectivity between the left and right prefrontal cortices, also important in the DMN and also observed to be affected in meditation practices (Garrison et al., 2015). In addition, parietal lobe changes have frequently been associated with other spiritual experiences such as absorption (Lifshitz et al., 2019) and self-transcendence, for example, in patients with lesions to this brain region (Urgesi et al., 2010).

Several regions of the temporal lobe, including the middle temporal gyrus and fusiform gyrus also were found to have altered functional connectivity in the female participants. These regions have similarly been found to be involved in various meditation practices.

In terms of sexual stimulation itself, one group (Georgiadis et al., 2006) studied the neurophysiological effects of clitoral stimulation using ^15^H_2_O PET imaging to measure changes in CBF. The results showed that sexual stimulation of the clitoris (compared to rest) significantly increased rCBF in the left secondary and right dorsal primary somatosensory cortex. Again, in the current study of the OM practice, we did not see changes in the somatosensory cortex, perhaps because in the OM practice, climax is not specifically achieved. In the study by Georgiadis et al. (2006), climax was mainly associated with profound rCBF decreases in the neocortex when compared with the control condition, particularly in the left lateral orbitofrontal cortex, inferior temporal gyrus and anterior temporal pole, which were not observed to have altered connectivity in the present study. Other studies have reported that climax in women is associated with activation of the cerebellum, anterior cingulate gyrus, and dopaminergic pathways, as well as the hippocampus and amygdala (Komisaruk et al., 2004). Of these structures, only functional connectivity with the amygdala and anterior cingulate was found to be significantly altered after the OM practice, but not in the females alone. It should be noted that our previous studies have found thalamic activation in a variety of meditation practices (Newberg et al., 2001, 2010b). The connectivity of the thalamus with the cerebellum was found to be significantly altered after the OM practice and since the thalamus is involved with sensory processing and the complex coordination of a variety of brain activities, it suggests that the practice is a highly active brain state.

Statistical results from that study revealed higher overall activity during orgasm than during cervical self-stimulation prior to orgasm (Komisaruk et al., 2004). The orgasmic response first activated the amygdala, basal ganglia (especially the putamen), and insula, followed by the cingulate cortex, and at orgasm, the nucleus accumbens, paraventricular nucleus of the hypothalamus, and hippocampus became activated. Finally, the orgasmic response was characterized by an overwhelmingly strong pattern of activation over a broad and distributed neural network including the prefrontal cortex, superior parietal region, and cerebellum. These findings support the hypothesis that an orgasm results from a spread of neural activation all over the brain, as suggested by the epileptic data we previously described. Shortly after the OM practice, there are alterations in the female participants in some of these areas such as the amygdala, cerebellum, and prefrontal cortex. However, the functional connectivity we observed does not comport with such a widespread process in the female participants, and the regions involved appear different including the thalamus, cerebellum, angular gyrus, and temporal lobe. These areas are found to be more specifically involved with meditation practices as described above.

With regard to males, genital stimulation has resulted in activation of midbrain, cerebellar, and dopaminergic areas along with a number of cortical regions (Holstege et al., 2003; Georgiadis et al., 2010). Temporal lobe and frontal lobe activity was reported to decrease at ejaculation in men, although middle temporal gyrus activity increased (Tiihonen et al., 1994). And activation was reported during ejaculation in the orbitofrontal cortex. Frontal cortical activation also was reported in women during orgasm, whereas deactivations of frontal cortical regions were reported based on PET studies and perfusion fMRI in men (Komisaruk and Whipple, 2005).

As with these findings in sexual climax, the current study of the OM practice did show significant changes in functional connectivity in the cerebellum and orbitofrontal cortex in the males, but most of the significant changes in functional connectivity were not in areas specific to orgasm. In the males, functional connectivity was altered in the supramarginal gyrus, orbitofrontal cortex, inferior temporal gyrus, and anterior cingulate gyrus. Again, these areas are described to be more involved in meditation based practices.

Interestingly, the males had altered functional connectivity in the supramarginal gyrus. This is consistent with our hypothesis that the males would have to orient themselves more specifically to the female participant. Since the supramarginal gyrus is involved with tactile sensory data, it would make sense that since the male is stimulating the female, he needs to be particularly attuned to how his own motor and sensory activity is facilitating an experience within the female. That the altered functional connectivity in the supramarginal gyrus is associated with the cerebellum and lingual gyrus, this further is consistent with the notion that this practice involves tactile stimulation combined with a strong social connection between participants.

One study differentiating orgasm from the emotion of love suggested that insular activity correlated with self-reported intensity of orgasm while angular gyrus activity correlated with the feeling of love (Ortigue et al., 2007). It is interesting in the present study that functional connectivity with the insula was observed only when the entire group of both males and females were analyzed. The female subjects had altered functional connectivity in the angular gyrus suggesting that this practice elicits more complex feelings than those associated with orgasm.

In comparison more generally to meditation studies, a variety of brain regions have been implicated in different elements of meditation and different aspects of the meditative experience. For example, a variety of studies have shown increased frontal lobe activity associated with attentional focus during meditation (Lenhart et al., 2020). The frontal lobe, and more specifically the attentional network which includes the lateral prefrontal cortex, premotor cortex, lateral parietal regions, occipital regions, anterior cingulate cortex, and insula, have been found to be activated during concentrative meditation techniques (Hasenkamp et al., 2012). We observed a significant change in functional connectivity in a number of these structures in both the male and female participants performing the OM practice.

In comparison to mindfulness practices, a randomized controlled trial, conducted by Wells et al. (2013), showed that a mindfulness-based stress reduction program which applied open monitoring techniques, resulted in an increased connectivity in the posterior cingulate cortex, medial prefrontal cortex, and left hippocampus in participants who suffered from mild cognitive impairment, as compared to controls who did not receive this intervention. Mindfulness practices also have also been shown to affect the salience network, the executive control network, and the orienting network (Sridharan et al., 2008; Malinowski et al., 2017). In the current study, we did not observe changes in functional connectivity in these regions, differentiating it from mindfulness itself.

In terms of intensity of the experience, all subjects taken together showed an association increased intensity of the OM experience and increased connectivity between the right frontal gyrus with the anterior cingulate and Heschl’s gyrus. Increased intensity of the experience was also associated with decrease connectivity between the cerebellum and nucleus accumbens. In females, intensity of experience was correlated with increased connectivity between the left cerebellum and the anterior cingulate/salience network. In males, intensity of the experience was correlated with increased connectivity between the right cerebellum with the precuneus and posterior cingulate/DMN, as well as the left temporal lobe and the left medial PFC/DMN. Intensity of the experience was also correlated with decreased connectivity between the left temporal lobe and the supramarginal gyrus and Heschl’s gyrus as well as the PFC and the precuneus.

These findings suggest that many of the areas associated with the OM practice are similarly associated with the intensity of the experience. It is interesting to note that the female participants had only one correlation between the intensity of the experience and the functional connectivity between the cerebellum and salience network. Alternatively, in the males, there are a number of structures involved with the intensity of the experience suggesting a more complex pattern. Perhaps the sexual stimulation in females leads to a relatively focused mechanism regarding the intensity of the experience that affects the salience network. However, since males are not specifically sexually stimulated, there are a number of brain regions that participate in making the connection between the male participant and the female partner.

In terms of our initial questions #3 and #4, we also found significant changes in autonomic activity based on heart rate variability data associated with the OM practice. These changes primarily reflected increased sympathetic tone. This finding is expected given the stimulatory nature of the practice and is consistent with findings of other meditation practices that involve intense focus resulting in increased sympathetic activity (Léonard et al., 2019; Telles et al., 2019). However, the data on heart rate variability in meditation practices is complex as many relaxation-based practices reduce sympathetic activity and augment parasympathetic activity (Olex et al., 2013; Tyagi and Cohen, 2016). Future studies will need to better clarify the relationship between heart rate variability and the diverse categories of meditation practices.

Furthermore, these autonomic changes were associated with functional connectivity changes on fMRI scans. Specifically, in all subjects, decreased RMSSD (associated with increased sympathetic tone) correlated with decreased functional connectivity between the right nucleus accumbens and right caudate and increased connectivity between the left parahippocampus and left precentral gyrus. In females, decreased RMSSD correlated with decreased functional connectivity between the left thalamus and right fusiform gyrus but increased connectivity between left and right structures such as the DMN and superior frontal gyrus and the dorsal attention structures and the putamen. And in males decreased RMSSD correlated with increased connectivity between the hemispheres such as the Right PFC and left opercular cortex, and the right cerebellum and left planum temporale. There was also a correlation with increased connectivity between the right supplementary motor cortex and right PFC and left precentral gyrus and left parahippocampus.

In all subjects there was a correlation of the high frequency power (also associated with increased sympathetic activity during OM) and increased functional connectivity between the left inferior frontal gyrus and the temporal and orbitofrontal cortices. There was a correlation between high frequency power and decreased connectivity in the vermis and right superior temporal gyrus and the right PFC and right Heschl’s gyrus. In females, there was a correlation between the high frequency power and increased functional connectivity between the vermis and left occipital and temporal gyrus, as well as the right cerebellum and the left central opercular cortex. There was a correlation between high frequency power and decreased functional connectivity between the right PFC and right putamen, left amygdala and right angular gyrus, and the right superior temporal gyrus and right and left hippocampus. In the males, the high frequency power correlated with increased connectivity between the left cerebellum and the right posterior parietal cortex and decreased connectivity between the right cerebellum and the temporal lobe.

The above findings indicate a combination of functional connectivity changes involving cortical and subcortical structures are associated with autonomic activity. These findings are consistent with the overall arousal state of the OM practice with decreased heart rate variability as measured by the decreased RMSSD and high frequency power. Further, it appears that increased sympathetic activity is associated with changes in brain regions that have an impact on overall arousal such as the thalamus, parahippocampus, prefrontal cortex, and dopaminergic structures of the nucleus accumbens and caudate. These results likely reflect increased sympathetic arousal as the result of the clitoral stimulation process which supports the notion that the OM practice uses this stimulation as the focus of the meditation. The correlation with cortical functional connectivity (e.g., various subregions of the frontal, temporal, and occipital lobes), further supports the notion that OM is a meditation practice which is, consistent with our initial hypothesis.

Limitations of the study primarily are associated with determining the best method for evaluating the participants during this practice. We selected BOLD fMRI, but the OM practice could not physically be performed in the scanner environment, and hence, all measures of functional connectivity are associated with changes to the brain after the practice was completed. However, while these results do not capture changes during OM itself, there are persistent significant changes in functional connectivity after the practice that might have implications for future therapeutic use of this practice for various psychological purposes. It should be noted, though, that the females and males were scanned in a sequential manner with the males scanned approximately 45 min later due to the logistics of putting subjects in the scanner. Several studies have indicated that the effects of a given task or activity will have an effect on resting BOLD data that will change depending on the delay (Miall and Robertson, 2006). That we were able to detect significant changes in spite of the differences in the delay between the OM practice and scan acquisition suggests that the effects are strong and persistent. But future studies focusing on one subject group, such as only the male or female subjects, would be required to better control the timing of the scans and assess changes over time in functional connectivity as the result of performing OM.

We randomized the ordering of the two conditions so that the findings are not associated with increased comfort of the subjects with the imaging procedure. The study paradigm regarding the OM and neutral conditions appears to have worked as we did not see significant changes in the primary sensory or motor areas since these were accounted for by having similar elements in both the meditation and neutral conditions. We did consider clitoral stimulation in both the meditative and neutral conditions, but the practitioners indicated that it would be too difficult not to do the actual practice if subjects were being actually stimulated. However, future studies might compare the brain changes of the practice more directly to other states including sexual stimulation and sexual climax, as well as other meditation based practices. Since this study was conducted with skilled OM practitioners, future research may include novice practitioners to see if the effect of change is consistent with these findings.

The findings of the study demonstrate specific patterns of functional connectivity associated with the OM practice and the types of spiritual experiences described by the participants. The findings also support the notion that both males and females are actively engaged in the practice and derive some similar experiences in terms of the meditative elements even though there are also distinctions between the male and female participants. These results are consistent with our initial hypotheses. Future research can continue to explore more specific neurophysiologic correlates and perhaps use other comparison states that include both meditative and or augmentation components. These results demonstrated that the OM practice has characteristics that appear to represent a unique hybrid between meditative practices and sexual stimulation.

These findings have implications for the broader topic of this special issue since there are important connections between sexuality and spirituality, as well as with creativity. These three human practices and activities are frequently intermingled and understanding how they may be related or linked physiologically is an important initial step in understanding this complex relationship. Further studies can utilize similar methods to more deeply explore these interdependent relationships.

## Data Availability Statement

The raw data supporting the conclusions of this article will be made available by the authors, without undue reservation.

## Ethics Statement

The studies involving human participants were reviewed and approved by the Thomas Jefferson University Institutional Review Board. The patients/participants provided their written informed consent to participate in this study.

## Author Contributions

ABN was involved with conceptualization, methodology, formal analysis, writing the manuscript, study supervision, and project administration. NAW was involved in project conception and development, study coordination, acquisition of data, data analysis, and writing the manuscript. CH was involved in study coordination, acquisition of data, data analysis, and writing the manuscript. FV was involved in acquisition of data, fMRI and data analysis, and writing the manuscript. MS was involved in acquisition of heart rate data, data analysis, and writing the manuscript. RR was involved in project conception and development, and writing the manuscript. All authors read and approved the final manuscript.

## Conflict of Interest

The authors declare that the research was conducted in the absence of any commercial or financial relationships that could be construed as a potential conflict of interest.

## Publisher’s Note

All claims expressed in this article are solely those of the authors and do not necessarily represent those of their affiliated organizations, or those of the publisher, the editors and the reviewers. Any product that may be evaluated in this article, or claim that may be made by its manufacturer, is not guaranteed or endorsed by the publisher.

## Footnotes

1
https://www.fil.ion.ucl.ac.uk/spm/
